# Weight bearing versus non-weight bearing ankle dorsiflexion measurement in people with diabetes: a cross sectional study

**DOI:** 10.1186/s12891-018-2113-8

**Published:** 2018-06-02

**Authors:** A. Searle, M. J. Spink, V. H. Chuter

**Affiliations:** 1 School of Health Sciences, Faculty of Health, University of Newcastle, PO Box 127, Ourimbah, NSW 2258 Australia; 2 Priority Research Centre for Physical Activity and Nutrition, University of Newcastle, Callaghan, NSW 2308 Australia

**Keywords:** Dorsiflexion, Ankle, Diabetes, Lunge, Reliability, Lidcombe

## Abstract

**Background:**

Accurate measurement of ankle dorsiflexion is important in both research and clinical practice as restricted motion has been associated with many foot pathologies and increased risk of ulcer in people with diabetes. This study aimed to determine the level of association between non-weight bearing versus weight bearing ankle dorsiflexion in adults with and without diabetes, and to evaluate the reliability of the measurement tools.

**Methods:**

One hundred and thirty-six adults with diabetes and 30 adults without diabetes underwent ankle dorsiflexion measurement non-weight bearing, using a modified Lidcombe template, and weight bearing, using a Lunge test. Pearson product-moment correlation coefficients, intraclass correlation coefficients (ICCs) with 95% confidence intervals, standard error of measurement and minimal detectable change were determined.

**Results:**

There was a moderate correlation (*r* = 0.62–0.67) between weight and non-weight bearing tests in the non-diabetes group, and a negligible correlation in the diabetes group(*r* = 0.004–0.007). Intratester reliability was excellent in both groups for the modified Lidcombe template (ICC = 0.89–0.94) and a Lunge test (ICC = 0.83–0.89). Intertester reliability was also excellent in both groups for the Lidcombe template (ICC = 0.91) and a Lunge test (ICC = 0.88–0.93).

**Conclusions:**

We found the modified Lidcombe template and a Lunge test to be reliable tests to measure non-weight bearing and weight bearing ankle dorsiflexion in adults with and without diabetes. While both methods are reliable, further definition of weight bearing ankle dorsiflexion normative ranges may be more relevant for clinical practice.

## Background

Ankle dorsiflexion is essential for normal gait, as the ankle first plantarflexes after heel strike to bring the forefoot into contact with the ground, and then dorsiflexes as the centre of gravity of the body moves over the joint during forward movement [1]. It is generally agreed that a minimum of 5–10° of ankle dorsiflexion is required for normal gait [2–4]. Equinus refers to restricted ankle dorsiflexion, and in the absence of a standardised definition [5], has been described as less than 10° [6, 7], less than 5° [3, 8, 9] or less than zero° of dorsiflexion [3, 10, 11]. An equinus can result from a number of causes including bony block, neurological abnormalities, soft tissue contracture resulting from prolonged inactivity or prolonged ankle plantarflexion, and metabolic changes common in aging and diabetes [12]. In the general population equinus has been associated with conditions such as chronic heel pain [13], Achilles tendonitis [14] and plantar fasciitis [15, 16]. In people with diabetes, equinus has been linked to increased ulcer risk [17] and delayed ulcer healing [11]. Therefore, accurate measurement of ankle dorsiflexion is important in both research and clinical practice to allow correct identification of restriction, and measurement of the efficacy of interventions to improve motion.

The most common measurement method reported is passive non-weight bearing ankle dorsiflexion using a goniometer, however major concerns have been raised about the reliability of this method [18, 19]. A modified Lidcombe template was developed to measure non-weight bearing ankle dorsiflexion and has been reported to have good reliability, but has not been tested in adult or diabetes populations [20]. Weight bearing ankle dorsiflexion, measured with an extended knee Lunge test has also been shown to be reliable in populations without diabetes, is practical for clinical use and may more accurately reflect restriction experienced during gait and activities of daily living [21]. If there is an association between the two measures, a Lunge test may be able to be recommended for use in clinical practice and research situations, while allowing comparison with previous results.

Therefore, the aims of this study were to test in adult populations with and without diabetes; 1) the reliability of a modified Lidcombe template to measure passive non-weight bearing ankle dorsiflexion, and, a Lunge test to measure weight bearing ankle dorsiflexion, and 2) the level of association between ankle dorsiflexion measured using the above methods.

## Methods

Ethics approval was granted by the University of Newcastle Human Research Ethics Committee and written informed consent was obtained from all participants. A group of 136 adults with diabetes were recruited from the University of Newcastle Podiatry Clinic at Wyong Hospital, NSW, Australia, and from newspaper advertisements in local newspapers, between June 2016 and October 2017. An additional 30 adults without diabetes were recruited from the student population of the University of Newcastle, Ourimbah, Australia. Inclusion criteria were adults, 18 years of age and over, able to provide informed consent and a diagnosis of either type 1 or type 2 diabetes for the diabetes group. Exclusion criteria were existing foot ulcer, wound or infection in the lower leg, any previous lower limb amputation, any surgery to the foot or lower limb involving fixation of a joint, any recent injury to the foot or ankle that may be exacerbated or result in pain due to movement of the ankle, and any problems that would prevent the participant from lying reclined for approximately 5 min.

### Procedures

To assess the reliability of the measurement techniques, 30 participants from each group were assessed by two testers at the same testing session on two separate occasions, five to ten days apart. This period of time has been used previously, and is considered to be sufficient to reduce recall bias of testers to previous results [22]. The order of testers was randomised to minimise bias from repeated testing. One podiatrist and one osteopath, both with 10 years of clinical experience performed all the measurements. Testing was conducted on the participants’ dominant leg only to maintain independence of data [23]. Dominance was determined by asking the participant which foot they would kick a football with. Participants in the diabetes group, who were part of an ankle equinus trial, were asked at the completion of their first appointment if they were available to attend another measurement session in one weeks’ time. Those who volunteered to attend two measurement sessions formed the diabetes reliability group.

**Fig. 1:**
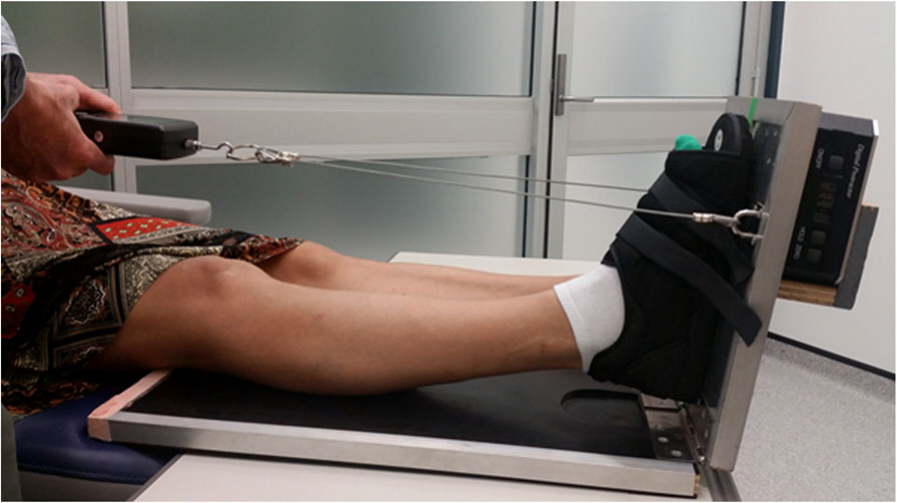
Lidcombe template

Non-weight bearing ankle joint range of motion was measured using a modified Lidcombe template which was designed to address the deficiencies in goniometric testing [20]. This consisted of a 300 mm solid foot plate hinged to a solid base plate (Fig. 1). A digital protractor (Bear Digital Protractor 82201B-00, China) was fixed to the back of the foot plate to allow the degree of dorsiflexion to be measured. To allow application of a consistent dorsiflexion force, a digital force gauge (FGD-200, Starr Instruments, Melbourne, Australia) was attached to the front of the foot plate at a distance of 200 mm from the hinge attachment. Participants were required to lay reclined with their knees extended on an examination table with the base plate of the device placed under the dominant leg. Participants were advised against actively dorsiflexing their ankle, flexing their knee or resisting the applied force during the examination. A standardised force of 80.4 N was applied to the base plate, by means of the tester pulling on the strain gauge, as this is believed to best replicate the forces experienced during gait [5]. The degree of dorsiflexion was read from the digital inclinometer by another investigator. The digital inclinometer was not visible to the tester which ensured blinding. The test was completed three times, with 10 s rest between tests, and the average score was documented as the test result.

**Fig. 2:**
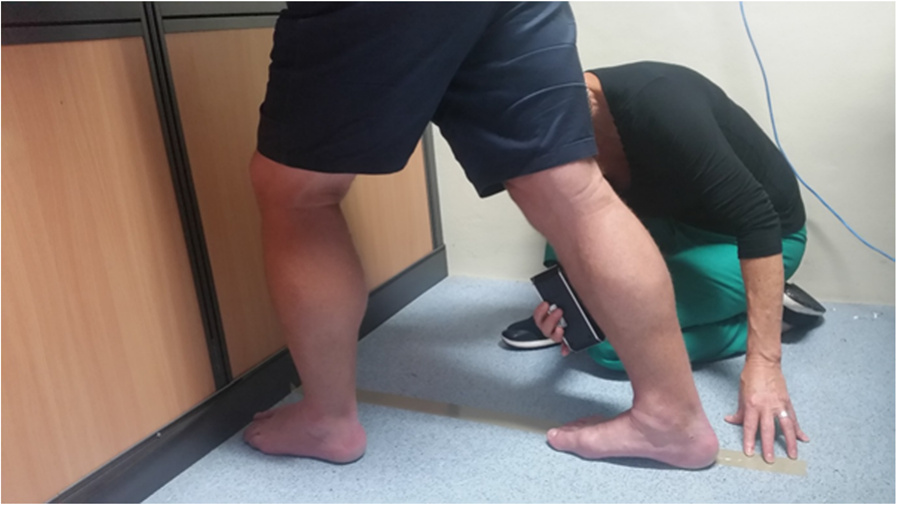
Measurement of ankle joint dorsiflexion using a Lunge test with knee extended

A Lunge test with the knee extended was used to determine ankle joint dorsiflexion range of motion in a weight bearing position (Fig. 2). This method was chosen as it has been reported to have excellent interrater (ICC = 0.82) and interrater (ICC = 0.88) reliability, and it measures the effects of gastrocnemius tightness, which is reported to be a main contributor to ankle dorsiflexion restriction [3, 24]. A tape line was placed on the floor perpendicular to the wall. The participant placed both hands on the wall in front of them and then positioned their dominant leg behind them as far as possible. The second toe and the centre of the heel of the participant’s dominant foot were situated over the tape line to minimise subtalar joint pronation during measurement. The participant was asked to lean forward until a maximum stretch was felt in the dominant leg, while keeping the heel of the dominant foot in contact with the ground, and the knee of the dominant leg fully extended. The digital protractor described above was placed on the midpoint of the anterior border of the tibia and the reading in degrees was recorded by the tester [21]. No pre-conditioning stretching was performed [25]. The test was completed three times, with 10 s rest between tests, and the average score was documented as the test result. Each tester read and recorded their own Lunge scores as would occur in clinical practice.

Neuropathy status was assessed using a monofilament and a neurothesiometer which are reliable tests for measuring foot sensation [26, 27]. Four points on the plantar surface of the dominant foot (1st, 3rd and 5th metatarsal heads and the distal hallux) were tested with a 10 g Semmes-Weinstein monofilament. An abnormal test was noted if the participant failed to identify the monofilament at one or more test sites [28]. A neurothesiometer (Horwell, Bailey Instruments, Manchester,UK) was used to detect the vibration perception threshold (VPT) at the pulp of the hallux. Three readings were taken and the average used in analysis. A VPT value of > 25 V was regarded as abnormal [28]. Participants were assessed as neuropathic if they recorded one or more abnormal test results.

### Sample size

For the measurement tool reliability calculations a sample size of 30 participants per group was determined based on α = 0.05, β = 0.20 for two observations per participant, with a target intraclass correlation coefficient (ICC) of 0.8 and a lowest acceptable ICC of 0.50 [29].

### Statistical analysis

All statistical tests were conducted using SPSS Release 24 for Windows (SPSS Inc., Chicago, Ill., USA*)*. Intra- and intertester reliability was assessed using interclass coefficients (ICC) and standard error of measurement (SEM) with 95% confidence intervals (CIs) and minimal detectable change (MDC). An ICC [1, 3] was calculated for intertester reliability between testers 1 and 2 in session one and intratester reliability between sessions one and two. Interpretation of ICCs was in accordance with Portney and Watkins [30]:> 0.75 = good, 0.5 to 0.75 = moderate, and < 0.5 = poor reliability. Measurement error was expressed in the original units using the standard error of measurement, SEM = SD*√(1-ICC) and the 95% CI = mean score ± 1.96(SEM) [30]. To determine the smallest amount of change in ankle dorsiflexion that must be achieved to reflect a true change, outside the error of the tests, the MDC was calculated as MDC_95_ = 1.96*SEM*√2 [30]. Paired t tests were performed for mean ankle dorsiflexion measures to determine whether a statistically significant difference existed for intratester scores between sessions 1 and 2 and for intertester scores in session 2 [30]. Differences in participant characteristics between the diabetes and diabetes reliability groups were evaluated by independent samples t-test for continuous variables and Chi-square test for categorical variables [30].

Pearson product-moment correlation coefficients were calculated to assess any correlation between a Lunge test and the modified Lidcombe template scores using the full diabetes group (*n* = 136) and the control group [30]. The Pearson r values were interpreted as follows: 0 to ±0.30 indicates a negligible relationship, ±0.30 to ±0.50 a low, ±0.50 to ±0.70 a moderate, ±0.70 to 0.90 a high and ± 0.91 to ±1.00 a very high relationship [31].

## Results

One hundred and thirty-six people with diabetes and 30 people without diabetes were recruited for the study (Table 1). The diabetes group were older, heavier, had a higher body mass index (BMI) and less weight bearing and non-weight bearing range of ankle dorsiflexion than the non-diabetes group (*p* < 0.01). There were no significant differences between the total diabetes group (*n* = 136) and the subset of participants in the diabetes reliability group (*n* = 30) for ankle dorsiflexion Lidcombe (*p* = 0.69), ankle dorsiflexion Lunge (*p* = 0.30), age (*p* = 0.33), BMI (*p* = 0.17), weight (*p* = 0.29), gender (*p* = 0.38) or neuropathy status (*p* = 0.15). Mean ankle dorsiflexion measurements did not differ significantly between session 1 and session 2 for either tester (Table 2).

**Table 1:** Participant Characteristics. Values are mean (SD) unless stated otherwise

	Diabetes group (*n* = 136)	Diabetes reliability group (*n* = 30)	Non-diabetes group (*n* = 30)
Age (years)	68.8 (10.7)	65.8 (16.0)	28.0 (6.8)
Age range (years)	25.6–91.8	25.6–86.5	21.7–55.9
Female (n)	65(47.8%)	17(56.7%)	14(46.7%)
Weight (kg)	92.9 (19.0)	97.2 (24.5)	78.0 (19.6)
BMI	32.7 (6.2)	34.5 (7.5)	25.6 (6.6)
Ankle dorsiflexion Lidcombe (degrees)	3.2 (5.8)	3.6 (6.7)	9.2 (5.8)
Ankle dorsiflexion Lunge (degrees)	33.0 (7.4)	34.5 (6.3)	39.8 (7.9)
Neuropathy (n(%))	65 (47.8%)	10 (33.3%)	0 (0%)

**Table 2:** Mean tester ankle dorsiflexion measurements and intratester *p* values in groups with and without diabetes. Values are degrees (SD)

	Diabetes reliability group (*n* = 30)			Non-diabetes group (*n* = 30)		
	Session 1	Session 2	p value	Session 1	Session 2	p value
Lidcombe template						
Tester 1	3.6 (6.7)	3.5 (6.8)	0.80	9.2 (5.9)	9.6 (5.0)	0.51
Tester 2	3.3 (5.9)	3.3 (6.5)	0.98	9.2 (5.8)	9.5 (5.3)	0.45
Lunge test						
Tester 1	34.5 (6.3)	34.2 (6.5)	0.66	39.1 (8.2)	39.1 (7.2)	0.97
Tester 2	34.4 (6.3)	35.1 (6.8)	0.30	40.6 (7.7)	41.5 (7.1)	0.17

### Intratester reliability

Intratester reliability for the use of the modified Lidcombe template to measure non-weight bearing ankle dorsiflexion in both diabetes and non-diabetes groups was excellent for both testers (Table 3). The ICCs ranged from 0.89 to 0.94 with 95% CIs from 0.78 to 0.97. Similarly, intratester reliability for the use of the Lunge test to measure weight bearing ankle dorsiflexion in both groups was excellent for both testers, with ICCs from 0.83 to 0.89 and 95% CIs from 0.67 to 0.94 (Table 3). The modified Lidcombe template SEM values were low, ranging from 1.6° to 2.0°, for both testers across the two groups, and the MDC were between 4.6° and 5.2°. The Lunge test SEM values were also low, ranging from 2.4° to 3.2°with MDC values between 6.2° and 7.6°.

**Table 3:** Intratester and Intertester reliability of ankle dorsiflexion measurements in groups with and without diabetes

	Diabetes reliability group			Non-diabetes group		
Measure	ICC (95% CI)	SEM (95% CI) degrees	MDC	ICC (95% CI)	SEM (95% CI) degrees	MDC
Intratester Reliability						
Lidcombe template						
Tester 1	0.94 (0.88–0.97)	1.6 (0.4–6.8)	4.6	0.89(0.78–0.95)	2.0 (5.4–13.0)	5.2
Tester 2	0.90 (0.80–0.95)	1.9 (−0.4–7.0)	5.1	0.89(0.78–0.95)	1.9 (5.5–13.0)	5.2
Lunge test						
Tester 1	0.83 (0.67–0.92)	2.6 (29.4–39.6)	6.5	0.85 (0.72–0.93)	3.2 (32.9–45.3)	7.6
Tester 2	0.85 (0.71–0.93)	2.4 (29.6–39.6)	6.2	0.89(0.78–0.94)	2.6 (35.6–45.6)	6.4
Intertester Reliability						
Lidcombe template	0.91 (0.83–0.96)	1.9 (− 0.2–7.2)	5.1	0.91 (0.81–0.95)	1.7 (5.8–12.6)	4.8
Lunge test	0.88 (0.77–0.94)	2.1 (30.3–38.7)	5.6	0.93 (0.85–0.96)	2.1 (35.7–43.9)	5.5

*Abbreviations*: *ICC* intraclass correlation coefficient, *CI* confidence interval, *SEM* standard error measurement, *MDC* minimal detectable change

### Intertester reliability

Intertester reliability for the use of the modified Lidcombe template to measure non-weight bearing ankle dorsiflexion in both diabetes and non-diabetes groups was excellent, with ICCs of 0.91 and 95% CIs from 0.81 to 0.96 (Table 3). Intertester reliability was also excellent for the use of a Lunge test to measure weight bearing ankle dorsiflexion in both groups with ICCs of 0.93 (non-diabetes group) and 0.88 (diabetes group) and 95% CIs from 0.77 to 0.96 (Table 3). SEM values for the modified Lidcombe template were 1.7° (non-diabetes group) and 1.9° (diabetes group), with MDCs of 4.8° and 5.1°. The Lunge test SEM values were also low at 2.1°, with MDC values of 5.5° and 5.6°.

### Correlations between weight bearing and non-weight bearing measures

There was a negligible correlation between the weight bearing and non-weight bearing measures of ankle dorsiflexion (*r* < 0.01, *p* = 0.99 and *r* = 0.01, *p* = 0.97 for testers 1 and 2 respectively) in the diabetes group. There was a moderate, significant correlation between the measures of ankle dorsiflexion in the non-diabetes group (*r* = 0.62 and *r* = 0.67 for testers 1 and 2 respectively, *p* < 0.01) in session 1. The weight bearing range of motion was approximately four times larger than the non-weight bearing motion in the non-diabetes group and approximately 10 times larger in the diabetes group (Table 1).

## Discussion

We found the modified Lidcombe template and a Lunge test to be reliable tests to measure non-weight bearing and weight bearing ankle dorsiflexion in adults with and without diabetes. The modified Lidcombe template showed excellent levels of intratester and intertester reliability. While this is the first study to assess the reliability of a modified Lidcombe template in people with diabetes, our findings are consistent with previously reported reliability in healthy participants and participants with pathology using both the original and modified Lidcombe templates. Moseley and Adams [32] and Keating et al. [33] both reported excellent intertester reliability of a Lidcombe template (ICC = 0.97 and > 0.92 to 0.97 respectively) in unimpaired adults, stroke impaired adults [32, 33] and those with head injuries [32]. Similarly Scharfbillig et al. [20] found excellent intratester and intertester reliability (ICC > 0.99) when using a modified Lidcombe template to measure ankle dorsiflexion in fourteen children aged 7 to 14 years.

Our Lunge test results also showed excellent intertester and intratester reliability for both the diabetes and non-diabetes groups. These results are consistent with a recent systematic review of reliability of the weight bearing lunge test in healthy populations [34]. The review found intertester reliability was excellent, ranging from ICC 0.80 to 0.99 across nine studies, while intertester reliability was reported as good to excellent, ranging from ICC 0.65 to 0.99 across twelve studies [34]. As 33.3% of the reliability group had neuropathy, and neuropathy is reported to affect 16 to 66% of people with diabetes [35], our results may be generalised to the wider diabetes population.

The high reliability of a weight bearing lunge test found in this study is particularly relevant to a clinical context. While ankle dorsiflexion measurement using the Lidcombe template addresses many of the known problems with non-weight bearing measurement, and we have shown it to be reliable in diabetes cohort and a healthy adult population, there are still limitations to its use in clinical practice. The device is not commercially available, there is a cost of acquiring the equipment and it requires two people for assessment. In contrast, a Lunge test requires only one individual with an inclinometer or smartphone [36], is fast, and may more accurately reflect restriction experienced during activities of daily living [37, 38]. Consequently an aim of this study was to determine any correlation between weight and non-weight bearing measures of ankle dorsiflexion, with an objective of being able to use a Lunge test in future practice while allowing comparison to past evidence.

Our results showed a moderate correlation (*r* = 0.62 and *r* = 0.67, *p* < 0.01) between weight and non-weight bearing measures of ankle dorsiflexion in the non-diabetes group, which is in line with the findings of other authors. Rabin and Kozol [39] also found a moderate correlation (r = 0.6 and *r* = 0.64, p < 0.01) between ankle dorsiflexion measured weight bearing and non-weight bearing in a group of 43 healthy young men and women (mean (SD) age and body mass: 25.5(4.9) years,63.3(12.2) kg). However, their non-weight bearing assessment was performed with a goniometer which has been shown to be unreliable [19, 40]. The authors found a similar moderate correlation (*r* = 0.61 and *r* = 0.55, *p* < .01) when they repeated the trial with 64 healthy young males (mean (SD) age and body mass: 19.6 (1.0) years,71.4 (7.7) kg) [41]. The moderate correlation may be a result of the two tests measuring different joint motions as some subtalar, midtarsal or tarsometarsal joint motion could be expected to occur in weight bearing when the foot is fully loaded [22].

In contrast, we found a negligible correlation (*r* < 0.01, *p* = 0.99 and *r* = 0.01, *p* = 0.97) between the two measures in the diabetes group. The heavier body weight in our diabetes group may have contributed to the differing results. During the stance phase of walking the ankle joint experiences forces of up to five times body weight, which in heavier people may be much larger than the standardised 80.4 N force applied in non-weight bearing [42]. In addition, non-enzymatic glycosylation occurring in diabetes and older age may have contributed to the development of stiffer tendon structures and increased resistance to the standardised force [12]. This is supported by the diabetes group having weight bearing measurements up to 10 times larger than the non-weight bearing measurements, compared to only four times larger in the non-diabetes group.

To achieve comparative results between the two tests it may be necessary to normalise the standardised force to body weight [8]. Alternatively, given the ease, high reliability and more functional position of weight bearing measurement [34], it may be more clinically relevant for further research to investigate a Lunge test in varied populations. This could provide a range of normative values for weight bearing ankle dorsiflexion and for ankle dorsiflexion restriction, similar to the zero and five degrees values that are in common use for non-weight bearing ankle equinus. A recent trial investigating standardised examination and normative values for weight bearing ankle dorsiflexion, proposed that in young healthy people values of < 30° with the knee extended should be considered impaired [43]. Our results indicate that 30.9% of our diabetes group and 16.7% of our non-diabetes group displayed weight bearing ankle dorsiflexion of < 30°.

MDC values were calculated for use in a clinical context such as before and after an intervention designed to increase ankle dorsiflexion range of motion. Average modified Lidcombe template MDC scores of 4.9° (diabetes group) or 5.2° (non-diabetes group) represent a clinically significant change in ankle joint motion and are well in excess of the SEM, indicating that such a change would be unlikely to be due to error and more likely to be due to an actual change in range of dorsiflexion. Keating reports similar figures of 7° for stroke impaired subjects and 3° for unimpaired students [33]. The average MDC scores for the Lunge test of 6.4°(diabetes group) and 7° (non-diabetes group) are slightly higher than the 4.7° reported in a review of healthy populations [34].

The results of this study need to be interpreted with the knowledge of its limitations. The tester was not blinded to the ankle dorsiflexion readings from the Lunge test, however, this is the method used in clinical practice. All the range of motion testing was conducted in one session which may have resulted in the muscle unit stretching during the session. This was mitigated by the rest period between readings, random allocation of the testers and averaging of the ankle range of motion results. Finally, the testers had practice sessions prior to the start of the trial to familiarise themselves with the Lidcombe template equipment therefore similar reliability may not be achieved by inexperienced testers.

## Conclusions

A modified Lidcombe template is a reliable tool for measuring non-weight bearing ankle dorsiflexion in both young adults and people with diabetes. A Lunge test is also a reliable test in these populations and, being weight bearing, is arguably a more functional measure of ankle dorsiflexion. A moderate correlation was found between the weight bearing and non-weight bearing measures in people without diabetes and the correlation was negligible in people with diabetes. Further investigation to define weight bearing ankle dorsiflexion normative ranges may prove to be more clinically relevant than refinement of non-weight bearing dorsiflexion assessment.

## Abbreviations

BMI: Body mass index
CIs: 95% confidence intervals
ICC: Intraclass correlation coefficient
MDC: Minimal detectable change
SEM: Standard error of measurement
